# Understanding factors influencing uptake of healthy lifestyle practices among adults following a community cardiovascular disease prevention programme in Mukono and Buikwe districts in Uganda: A qualitative study

**DOI:** 10.1371/journal.pone.0263867

**Published:** 2022-02-17

**Authors:** Rawlance Ndejjo, Geofrey Musinguzi, Fred Nuwaha, Hilde Bastiaens, Rhoda K. Wanyenze

**Affiliations:** 1 Department of Disease Control and Environmental Health, School of Public Health, College of Health Sciences, Makerere University, Kampala, Uganda; 2 Department of Family Medicine and Population Health, Faculty of Medicine and Health Sciences, University of Antwerp, Antwerp, Belgium; All India Institute of Medical Sciences Bhopal, INDIA

## Abstract

**Introduction:**

Healthy lifestyle practices including physical activity, healthy diets, non-smoking, reduced alcohol consumption and stress reduction are important in the prevention of metabollic CVD risk factors such as hypertension, overweight and obesity, diabetes and hyperlipidaemia. Owing to current lifestyle changes, the increasing burden of CVD and importance of healthy behaviours, the need for strategies to increase uptake of healthy lifestyles among sub-Saharan African populations are apparent. This study explored the factors influencing uptake of healthy lifestyle practices among adults following implementation of a community CVD prevention programme.

**Methods:**

This was a descriptive qualitative study conducted among purposively selected adults who had engaged in a community CVD prevention programme. Data were collected using in-depth interviews, which were audio recorded and transcribed verbatim. Study transcripts were read into NVIVO 12.6 software for coding and analysis guided by thematic analysis following the semantic approach.

**Results:**

This study found variations in uptake of healthy lifestyle practices for CVD prevention with most changes reported for dietary behaviour especially in vegetable and fruit intake, reduction of salt intake and fats and oils consumption. Changes in physical activity were also notable. On the other hand, participants were slow in making changes in alcohol consumption, smoking behaviours and stress reduction. The barriers to uptake of healthy lifestyle practices were individual such as limited capability or skills, structural such as limited physical activity facilities, and social such as cultural and peer influence. Relatedly, the facilitators of practices uptake were individual including knowledge and personal determination to change, and social including social support from family and the community.

**Conclusions:**

Insights from understanding the uptake of lifestyle practices should guide planning and design of community programmes with an emphasis on removing barriers and strengthening facilitators building on the intermediate motivating factors and considering individual needs and expectations.

## Introduction

Non-communicable diseases (NCDs) are increasingly becoming prevalent in sub-Saharan Africa (SSA) due to a shifting disease burden from mostly communicable to both infectious and chronic diseases [1]. Cardiovascular disease (CVD), the leading cause of death globally, is responsible for over one million deaths in SSA annually [2], expected to rise along with the projected increase in NCDs burden. The prevalence of hypertension, the leading risk factor for CVD, is also very high in SSA at over 30% among adults [3, 4]. In Uganda, of the 33% deaths due to NCDs overall, 10% are attributed to CVD [5]. Moreover, more than 25% of adult Ugandans are estimated to have hypertension though levels of status awareness and knowledge are low among the population [6–8]. The prevalence of other metabolic risk factors such as obesity and diabetes is also rising in Uganda [9, 10].

Healthy lifestyle practices including physical activity, healthy diets, non-smoking, reduced alcohol consumption and reduced stress are important in the prevention of metabolic CVD risk factors such as hypertension, overweight and obesity, diabetes, and hyperlipidaemia [11]. For example, leisure-time physical activity [12–14] and healthy diets such as consumption of fruits and vegetables [14–16] and lower salt intake [14, 16, 17] are associated with reduced CVD risk and mortality. Moreover, smoking [18] and alcohol consumption increase CVD risk and mortality [19, 20]. Therefore, by targeting behavioural risk factors and facilitating uptake of healthy lifestyle practices, the CVD burden can significantly be reduced [11].

Owing to current lifestyle changes in SSA and the importance of healthy behaviours, the need for strategies to increase uptake of healthy lifestyles among the populations is apparent. The World Health Organization through its global action plan for prevention of NCDs targets to increase physical activity levels by 10% and achieve a 30% relative reduction in mean population intake of salt/sodium [21]. The other targets are: a 30% relative reduction in prevalence of current tobacco use and a 10% relative reduction in harmful alcohol use by 2025 [21]. The same report targets to obtain a global relative reduction in the prevalence of raised blood pressure of 25% and premature mortality from NCDs [21]. Achieving these targets would contribute to attaining target 3.4 of the Sustainable Development Goals to reduce by one-third premature mortality from NCDs by 2030 [22].

The potential of community-based interventions to contribute to NCDs prevention and control has been previously elaborated in low- and middle-income countries and a few studies highlighted their effectiveness on CVD metabolic risk factors [23–26] including a recent systematic review [27]. These studies have also highlighted changes in some individual CVD prevention behaviours [23–27]. However, an in-depth understanding of factors that influence changes in individual CVD related behaviours remains limited which is critical in informing intervention design and implementation of community-based strategies for impact. Thus using in-depth interviews, this study explored the factors influencing uptake of healthy lifestyle practices among adults following implementation of a community CVD prevention programme. The community programme was implemented under the Horizon 2020 funded Scaling-up Packages of Interventions for Cardiovascular disease prevention in selected sites in Europe and sub-Saharan Africa (SPICES) project [28] implemented in Mukono and Buikwe districts of Uganda. The programme had community health workers (CHWs) working with existing community networks and structures to conduct CVD risk assessment and promote knowledge, improved lifestyles and cardiovascular health [28]. Specifically, the programme involved training and empowerment of CHWs to lead CVD prevention and control activities within their communities. The CHWs conducted house-to-house visits in the community to screen for CVD risk factors using the INTERHEART non-laboratory tool–a CVD risk assessment tool based solely on clinical history and simple physical measurements [29]. CHWs also provided health education and promoted lifestyle change through motivational interviewing and goal setting techniques [28, 30]. In doing the motivational interviewing, the CHWs used the 5As (Ask, Advise, Assess, Assist and Arrange) approach [31] focusing on emerging risks from the INTERHEART screening and worked with community members to create a roadmap to change the behaviour. Additionally, CHWs referred high-risk individuals to health facilities and followed them up in the community [28].

The community CVD prevention intervention was based on the Self Determination Theory (SDT) and the principles of motivational interviewing. The SDT is a theory of personality development and self–motivated behaviour change while motivational interviewing is a counselling style for facilitating behaviour change and the two are complementary [32, 33]. The SDT highlights behaviour change as a function of individual differences in motivational orientations, contextual influences on motivation, and interpersonal perceptions. According to the SDT, individuals will engage in an activity when intrinsically motivated to experience enjoyment or excitement, exercise their skills and for personal accomplishments [34]. Conversely, those externally motivated would do so to obtain an outcome different from the activity such as a social reward or avoiding disapproval [34]. The theory further highlights three basic psychological needs that influence satisfaction and autonomous motivation. These are i) competence–confidence and competence to change or control the outcome and experience mastery; ii) autonomy–control individuals have to engage in behaviour including the source of motivation; iii) relatedness–connectedness and trust with people more likely to adopt values and behaviours promoted by those they trust or feel connected [34]. The SDT has previously been applied to understand CVD related behaviours including physical activity, diet and smoking cessation [35–37].

## Methods

### Study area

This study was conducted in Mukono and Buikwe districts in Uganda where the SPICES project was being implemented targeting 20 parishes that were grouped into four clusters. Mukono and Buikwe districts have a population of 1,000,000 persons, 70% of whom reside in rural areas, engaging mostly in subsistence agriculture and fishing, while others in semi-urban areas operate small businesses in trading centres [38]. The study was conducted in five parishes that had received the intervention for one year as they were in the first cluster of the stepped wedge randomised design of the project [28]. Within the parish, the intervention was implemented in four randomly selected villages led by CHWs. In the Ugandan context, CHWs are volunteers with the ability to read and write in the local language and are selected by their communities to link them with the health system [39].

### Study design and population

This was a qualitative descriptive study that involved in-depth interviews with adults who had received the SPICES project intervention to explore their uptake of healthy lifestyle behaviours for CVD prevention. Implementation of the project interventions started in January 2019 and evaluation occurred in August 2020. The study participants in the five parishes had at least received a visit from a CHW who discussed CVD prevention measures with them and their INTERHEART scores taken. The selection of the study participants was purposive using the collected INTERHEART screening data containing age, gender and village of respondent, and scores on different health behaviours including smoking, physical activity, and diet. The selection process ensured diversity in age (less or equal to 40 vs above 40 years), gender (male vs female), parish (Katoogo, Misindye and Nabalanga in Mukono district and Ssi bukunja and Busabaga in Buikwe district) and scope of health behaviours especially for smoking and alcohol which applied to only a few. Physical activity, diet and stress were explored across all selected participants. At first, 36 participants were sampled of whom only 20 were traced and willing to participate. Thereafter, an additional list of 20 participants was sampled considering the characteristics of already included participants. Of these 11 participants were traced and interviewed and saturation of data was obtained. The study participants who could not be traced were either not found at home on at least three visits, not within their villages during the fieldwork, or only returned home late in the evening.

### Data collection

To obtain data for this study, participants were interviewed from their homes while ensuring privacy. The in-depth interviews were conducted using a pretested guide (S1 File) that had general questions on CVD, lifestyles changes before and after CHW visit, factors influencing uptake of any behaviours and any benefits of changes undertaken. The interview guide was translated into the local language, *Luganda*, in which the interviews were to be done. An independent team also supported the validation and revision of the translated interview guides before starting data collection. Interviews lasted on average 45 minutes (range 35 to 60 minutes) and were conducted by a research team (including the first author) composed of graduates in health or social science studies with experience conducting qualitative research and fluent in *Luganda*. The research team was composed of three males and three females who individually conducted the interview and took notes. The research assistants received a two-day training on conducting qualitative interviews and were introduced to CVD prevention and the interview guide. The interviewers were not personally known to the community members whom they contacted directly guided by name and location data collected during the INTERHEART screening without involving CHWs. The in-depth interviews allowed describing individual experiences and behaviours [40].

### Data management and analysis

All interview audio recordings were transcribed verbatim and concurrently translated into English by the research team. Thematic analysis of the data was conducted following the semantic approach [41]. Specifically, two authors read through selected study transcripts and developed an initial codebook based on the interview guide which they discussed and harmonised. All transcripts were then read into NVIVO 12.6 software and coding of the transcripts done across specific behaviours with any emergent codes included. Codes across the different health behaviours were grouped to form sub-themes in line with the study’s themes. Reporting for the study was supported by the Consolidated Criteria for Reporting Qualitative Research guidelines [42] and typical quotations are provided to support study findings.

### Ethical considerations

This study obtained ethical approval from the Higher Degrees Research and Ethics Committee of Makerere University School of Public Health (protocol 624) and was registered by the Uganda National Council for Science and Technology (HS 2477). Permission to undertake the study was obtained from the district authorities. Study participants provided written informed consent and their privacy and confidentiality were ensured.

## Results

### Participant characteristics

A total of 31 community members participated in this study, 16 of whom were female and 13 were aged between 20 to 40 years. Most participants had attained primary education (22), were married (21), and engaged in farming (22) (Table 1).

**Table 1:** Characteristics of study participants.

Characteristic	Number of participants (n = 31)
**Sex**	
Female	16
Male	15
**District**	
Mukono	19
Buikwe	12
**Age group (years)**	
20 to 40	13
41 to 67	18
**Education**	
None	3
Primary	22
Secondary	5
Tertiary	1
**Marital status**	
Single	2
Married	21
Separated/ divorced / widowed	8
**Occupation**	
Farming	22
Business	5
Other (Teacher, casual labourer, housewife)	4

The study results are presented under two domains: uptake of healthy lifestyles following the community programme which are described basing on the individual behaviours and factors influencing uptake of healthy lifestyles highlighted across the four identified themes.

### Uptake of healthy behaviours following the community programme

Following the community CVD prevention programme, community members reported about the changes they made across five behavioural risk factors of: diet, physical activity, alcohol use, smoking and stress.

#### Diet

Among our study participants, 27 reported changes in elements of their diet following the intervention. The most reported changes were in increased vegetable consumption (24) and fruit intake (13), reduced salt (14) and sugar consumption (3), and reduced fat intake (15).

#### Physical activity

Nineteen of the study participants reported changes in physical activity due to the intervention. The reported changes were in duration, intensity and variety of activities such as running, playing sports and engaging in domestic work.

#### Alcohol use

Nine of our study participants reported consuming alcohol among whom one stopped completely and six reported reductions in the amount, volume and frequency of their intake.

#### Smoking

Among our study participants, five (males) were tobacco smokers among whom only two reported changes in their smoking behaviour with one ceasing smoking and the other reducing the amount smoked. Two of the smokers had quit smoking cigarettes mainly due to prohibitive costs and resorted to crude locally grown tobacco.

#### Stress

Fourteen of our study participants reported making changes to reduce stress or avoid stressful events due to their awareness of it being a risk factor for CVD and its undesirable effects on their health. The key sources of stress were related to family, relationships and having limited resources to provide for themselves and their families.

### Factors influencing uptake of healthy lifestyle practices among participants

Four themes explained the factors that influenced uptake of healthy behaviours among study participants. These were: motivation to change, benefits of change, and barriers and facilitators of adopting the practice. These themes together with the subthemes are summarised by practices in Table 2 and are expounded below supported by individual quotations.

**Table 2:** Summary table of study themes and sub themes overall and across health behaviours.

Theme	Sub-themes	Diet	Physical activity	Alcohol	Smoking	Stress
**Motivation to change**	Individual factors (awareness and knowledge, and CVD risk perception)	Improved knowledge regarding CVD and its prevention. High CVD risk perception	Improved knowledge regarding importance of physical activity. Desire to reduce CVD risk	Awareness of harmful effects of alcohol consumption.	Obtaining information on harmful effects of passive and active smoking.	Awareness of stress being a risk factor for CVD. Undesirable effects on their health.
	Anticipated benefits (health and financial)	Desire to live a healthy lifestyle. Control of underlying health conditions.	Desire to be healthy. Reduced difficulties undertaking usual activities.	Desire to reduce expenditure on alcohol.	Smoking being expensive to maintain.	
	Social factors	Influence of significant others such as friends.	Influence by others especially peers.	Negative effect of alcohol on their social lives.	Effect of smoking on social life including relationships.	
**Benefits of change**	Health benefits	Improved health and wellbeing (increased body strength, reduced illness, loss of weight, improved heart function). Control of underlying health conditions.	Improved health and wellbeing (physical fitness, maintaining a healthy weight or feeling lighter, relief of stress and other ailments, increased strength and energy). Managing underlying health conditions.	Improved health and wellbeing. Increased energy to participate in day to day activities including sports.	Improved health and wellbeing (increased body strength and reduced coughing).	Maintaining better health and wellbeing including having better quality sleep. Having a more positive outlook to life and being happy.
	Social benefits			Respect from the community.	Maintaining social relationships.	
**Barriers to practice**	Individual barriers	Limited skills in food preparation. Being used to salty and oily foods. Limited financial resources to purchase fruits and vegetables.	Underlying conditions or illness such as heart conditions. Time constraints to engage in physical activity.	Not experiencing any harmful health effects from alcohol use. Alcohol as avenue to deal with stress and loneliness or relax and get sleep.	Not experiencing any health problems from smoking. Role of smoking in dealing with stress and boredom and a source of courage for intense manual work. Perceiving tobacco as less harmful than cigarettes.	Gaps in how to deal with stress or stressful events. Low incomes and daily struggles to obtain necessities of life.
	Structural barriers	Limited access to vegetables and fruits especially in rural areas. Fruits and vegetables being seasonal with reduced availability during the dry season. Limited ability to grow own vegetables and fruits due to lack of land or fruits taking a long time to grow.	Sometimes unfavourable weather for outdoor activities. Reduced work and sports related activities due to COVID-19. Limited variety of activities to engage in due to lack of resources including space.		Easy accessibility to tobacco which they mostly grew in their gardens.	
	Social barriers	Food served at communal events or public places such as restaurants being prepared differently.	Cultural related factors such as it not being acceptable for adults to jog. Spousal restrictions especially around places the other can go to. The lack of role models or peers for inspiration.	Peers attracting those making changes to going back to drinking places.		
**Facilitators of practice**	Individual (personal determination, knowledge and skills, integrating behaviour into lifestyle)	Personal determination to change and sustain behaviour and benefits. Ability to grow own vegetables and fruits or purchase them including during the off season. Adapting to healthy dietary practices including preparation of own meals. Considering diet as remedy for other underlying conditions. Attending training on how to grow vegetables including in small spaces. Frequent sensitisations or reinforcement of messages by health workers.	Incorporating physical activity in routine schedules or usual activities. Determination to keep healthy. Engage in light exercises or usual activities such as farming without intentional planning. Engaging in culturally appropriate physical activities.	Personal willingness and determination to change and its benefits. Desire to save resources they were committing to alcohol. Engaging in intense manual work which discouraged regular drinking.	Personal determination to change. Acknowledging harmful effects of smoking on health. Engaging in less manual intense work. Engaging in other activities.	Obtaining similar information on the dangers of stress from health workers Engaging in physical activity and prayers. Reducing workload or working for reduced hours. Engaging in distractive activities such as work Embracing self-counselling to keep on track.
	Social factors	Family support and commitment.	Forming physical activity groups especially among the youths to play sports or run together.	Avoiding peers to keep up with their changes in behaviour. Regaining community respect.	Support to change from family and peers.	Sharing challenges with colleagues. Spending time with peers.

### 1. Motivation to change

Three sub-themes explained motivation to change behaviour. These were: individual factors relating mostly to knowledge, awareness and risk perception; anticipated benefits especially in improved health or financial savings; and social factors driven by those in their circle of influence or community.

#### Individual factors

Many study participants were motivated to change their behaviours due to their improved awareness and knowledge regarding CVD and its prevention especially the harmful health effects of previous behaviours. Indeed, obtaining information from CHWs about the harmful effects of poor diets, physical inactivity, alcohol intake and smoking, and becoming aware that stress was a risk factor for CVD motivated change.

*“Before the sensitisation I used to take about four months without eating vegetables but when I was visited by the CHWs*, *we were encouraged to eat fruits and vegetables and I now do so at least twice a week*.*”*

(Male, 30 years)

*“Yes*, *I sometimes worry about things in life but I also counsel myself not to worry so much because I know it can lead to heart disease*.*”*

(Female, 33 years)

Those who were interested in changing dietary and physical activity behaviours also attributed it to their perception of having a high CVD risk and their desire to reduce it.

“*When they [CHW] first measured my weight*, *I was slightly heavy and they cautioned me that I might get high blood pressure*. *By that time*, *I wasn’t engaged in any active work but I decided to start right away*. *The CHW had advised me to engage in active work and eat vegetables more often to prevent CVDs*.”

(Female, 40 years)

#### Anticipated benefits

Community members anticipated benefits from changing behaviours which influenced their motivation to change. The major anticipated benefits were the desire to live a healthy life while others highlighted the need for healthy behaviours to support their control of underlying conditions.

“*We want to live healthy lives and good enough we are not buying it with money*. *You thus make sure you devote yourself as a person to remain healthy which is much easier than seeking treatment afterwards*.*”*

(Male, 41 years)

For alcohol intake and smoking, the other motivation was the desire to reduce expenditure on alcohol and tobacco to cater for other responsibilities as the behaviours were expensive to maintain.

*“In the past*, *I used to drink almost daily*, *and you would have found me drunk at this point*. *However*, *following understanding how alcohol was affecting my finances*, *I got fed up as it was not okay for me to be buying it every now and then*, *yet I had other family responsibilities*. *In fact*, *as I reduced on alcohol intake*, *I started feeling better and the information from our CHW has helped to keep me going*.*"*

(Male, 45 years)

#### Social factors

Social pressure such as the influence of significant others especially family and peers also influenced the motivation to change unhealthy behaviours for diet, physical activity, smoking and alcohol consumption. Indeed, community members who desired to stop smoking or reduce alcohol consumption reported negative experiences from their behaviours which had impacted their social lives including relationships and sometimes led to rebuke by other community members.

*“You may get too drunk and end up falling on the roadside and people will say so and so’s husband is a drunkard whose life depends on alcohol*. *Such ridicule from the community and the pressure from your wife together with regular education sessions is sufficient for you to stop drinkin*g.”

(Male, 34 years)

### 2. Benefits of change

Community members who reported changes in health behaviour majorly reported health benefits including weight reduction, improved heart function and wellbeing, or social benefits such as community respect and better relationships.

#### Health benefits

Participants mentioned health benefits following their uptake of healthy behaviours mostly reporting improved health and wellbeing for all behaviours. For changes in dietary behaviour, increased body strength, reduced frequency of illness, loss of weight, improved heart function and control of underlying health conditions were highlighted. On the other hand, changes in physical activity resulted in health gains and physical fitness, increased strength and energy, maintenance of a healthy weight, relief of stress and control of underlying health conditions.

*“Ever since I started eating vegetables following advice by the CHW*, *I feel better*, *and most of all*, *I am no longer feeling sickly*. *When you are eating vegetables*, *it is rare to fall sick as your body can fight against illnesses*.*”*

(Male, 24 years)

“*In the past*, *I could not climb a hill because my heart could start racing so fast*, *but ever since I started undertaking the physical activity measures advised by the CHW*, *this problem stopped and I started feeling better*. *Previously*, *I could feel too heavy but nowadays I am light*.”

(Female, 28 years)

The reported benefits in changing alcohol consumption patterns or behaviour were increased energy to participate in day to day activities including sports. Among smokers who ceased smoking or reduced the amount of tobacco smoked, the reported health benefits were increased body strength and reduced coughing. The benefits in reducing stress were related to having better quality sleep, a more positive outlook on life and being happy.

*“If I don’t get worried and stressed*, *I am at peace with myself and happy*.*"*

(Female, 36 years)

#### Social benefits

Besides health, there were social benefits due to abandoning alcohol or ceasing smoking reported by community members. These were largely around regaining community respect and maintaining social relationships which had previously been negatively impacted.

*“I used to play football as well as ride a bicycle but with time*, *I started struggling to participate in these activities as I could get extremely exhausted*. *My friends said it was alcohol weakening my body*. *Of recent*, *I drink only occasionally and I am slowly regaining respect from the community and my friends*.

(Male, 34 years)

### 3. Barriers to uptake of practice

The barriers to uptake of healthy lifestyle practices for CVD prevention were individual in terms of limited knowledge and skills, structural including the lack of a supportive environment or social including negative influence of peers or the community.

#### Individual barriers

Individual barriers in terms of knowledge and skills such as in food preparation or dealing with stress, underlying health conditions impacting doing physical exercises, limited financial and time resources including to purchase fruits and vegetables or to engage in physical activity affected uptake of behaviours. Specifically, for dietary practices, limited access to vegetables and fruits especially in some rural areas, the limited varieties grown and fruits and vegetables being seasonal–harder to find during the dry season–were barriers.

*“You have to plant vegetables in the rainy season*. *I can show you my garden where I planted Nakati (local vegetable—Solanum Aethiopioum) but now it looks miserable because of the sun*. *Time reaches when you don’t have vegetables and you just have to buy from the market and yet you may not have the money*.*”*

(Female, 65 years)

*“The CHWs and health workers simply told me to stop worrying and avoid being stressed*. *I have however not received information on how best to avoid stress and neither have I undergone any training on this*.*”*

(Female, 40 years)

Among those who consumed alcohol or smoked, they reported not experiencing any negative health effects or that the practice supported them to deal with stress and boredom or to effectively carry out their work making them reluctant to change. Most of those who smoked used tobacco which they perceived to be less harmful than cigarettes further hindering change.

“*We usually take alcohol because of the circumstances*. *As a poor man living in this small house alone*, *when I am bored and in deep thought*, *I am forced to get some alcohol to help me have peace of mind*.”

(Male, 60 years)

“*It is possible to quit tobacco smoking but because of the nature of the work that I do*, *I sometimes need to smoke some tobacco to get the energy and courage to go about my hard work deep down in the forest*.”

(Male, 38 years)

#### Structural barriers

Structural barriers were reported mainly for adopting changes in dietary practices and physical activity. Some community members for example mentioned that they did not have sufficient land to grow fruits and vegetables as they were tenants and also that fruits took a longer time to grow. Moreover, public places including restaurants did not prepare healthy meals impacting behaviour change strides. Relatedly, community members reported that they lacked physical activity facilities limiting the activities they could engage in. Moreover, the weather sometimes was noted not to be favourable for outdoor activities while the impact of COVID-19 restrictions also reduced the work and sports-related activities.

*“I used to be part of the women’s team that plays netball and encouragement from the CHW helped us to continue going but we have been unable to continue due to the current situation of COVID-19*. *We could exercise for one and a half hours which was adequate time for us but not anymore*.*”*

(Female, 42 years)

Regarding smoking, community members noted that the easy access to tobacco that was locally grown hindered their cessation efforts.

#### Social barriers

Community members reported social barriers in uptake of healthy CVD prevention practices. For physical activity, culture-related factors such as it not being acceptable for adults to jog which is considered funny or women to ride bicycles which is ostracized, and the lack of role models or peers to inspire others were noted. For dietary practices, food choices served at communal events or gatherings was noted not to be healthy. The barriers in adopting changes in alcohol consumption were peers who sometimes still attracted those making changes to drink especially when they go around drinking places.

“*Some of my former drinking peers could ask why they had not seen me in a long time and I would lie to them that work has been busy even when this may not be the case*. *At the back of my mind*, *I know setting my foot where they drink from will mean spending all my savings*.”

(Male, 34 years)

### 4. Facilitators of uptake of practice

As with barriers to practice, similar individual and social factors facilitated the uptake of healthy lifestyles for CVD prevention.

#### Individual facilitators

The key facilitators for change across all behaviours were the individual willingness and determination to change and having the requisite knowledge and skills which was through interaction with CHWs, health workers or attending training. With such knowledge and skills, community members were able to grow vegetables and fruits including in small spaces and/or prepare their meals. To keep up with increasing their physical activity levels or patterns, participants reportedly incorporated it into their schedules or usual activities as a routine sometimes doing it first thing in the morning or late in the evening and engaging in culturally appropriate physical activities.

*“We were trained on how to set up vegetable gardens in very small spaces*. *Even when you are a tenant*, *you can plant the vegetables in sacks and place them along the house veranda to grow*. *Also*, *some vegetables can be planted in buckets or a 10-litre jerrycan cut at the top and you hang them under a house shade to grow*.*"*

(Female, 42 years)

For alcohol consumption, smoking and stress reduction, adjusting their workload, schedules or work composition were facilitators. In addition, those who reduced their alcohol consumption saved resources, which they liked, while engaging in physical activity and other distractive activities including work supported stress reduction.

*“I enjoy football and in most cases*, *it takes away my stress because you come back from playing when tired and only get to rest*.*”*

(Male, 28 years)

#### Social factors

Among the social factors, family, peers and community support were important in facilitating behaviour change. For diet, participants noted that sharing information with their family who sometimes prepared the meals was beneficial while for physical activity, community members especially youths formed physical activity groups to play sports or run together.

***“****Educating the people I stay with has helped me to stay committed to a healthy diet and keep me in check*. *The good relationship I have with my wives and children has also helped because they are the ones that prepare the food while I am off to look for money*.*”*

(Male, 45 years)

“*We have colleagues with whom we studied*, *about 30 of us*, *and four are family members*. *So*, *when we are at home*, *we say that it is time for physical activity*, *then we go and do it*. *We are like family here*.”

(Male, 24 years)

On the other hand, participants who made changes in alcohol consumption reportedly avoided their peers to keep up with changes in behaviour. To support stress reduction, participants shared their challenges with colleagues and engaged in distractive activities such as work or spending time with peers. One community member also reported quitting smoking due to influence from a significant other.

“*I had been educated about the harmful effects of smoking but I had not taken any steps to stop it*. *I later quit smoking because my wife didn’t like it*. *So*, *to keep the relationship*, *I had to quit smoking*.”

(Male, 58 years)

## Discussion

This study explored uptake of healthy lifestyle practices following implementation of a community CVD prevention programme in Mukono and Buikwe districts in Uganda. The study found variations in uptake of healthy practices with most changes reported for dietary behaviour especially in vegetable (24/31) and fruit intake (13/31), reduction of salt intake (14/31) and fats and oils consumption (15/31). Changes in physical activity were also notable (19/31). On the other hand, participants were slow in making changes in alcohol consumption (7/9), smoking behaviours (2/5) and stress reduction (14/31). Community-based interventions have potential to influence changes in CVD-related behaviours including dietary practices, physical activity, smoking and alcohol use as has been previously reported [43, 44].

The motivations for uptake of healthy behaviours were related to knowledge and individual CVD risk perception, social influence and anticipated benefits of the change similar to previous research [45, 46]. The major benefits participants derived from changing behaviour were improvements in physical and psychosocial health and wellbeing, and social relations, which are key motivations for behaviour change per a recent review [45]. Barriers to uptake of healthy lifestyle practices were related to limited capability or skills, limited resources, accessibility challenges, low risk perception and effects of COVID-19. On the other hand, the facilitators of practices uptake were knowledge, skills and personal determination, integration of behaviour into lifestyle, and social support. A previous review of barriers and facilitators of uptake and maintenance of healthy behaviours reported a lack of knowledge, time, finances, entrenched attitudes and behaviour, and access issues as barriers [45]. Conversely, the same review highlighted the integration of behaviours into lifestyles, health benefits including healthy ageing, social support and clear messages as facilitators [45]. The role of personal determination and self-efficacy [47] and social support are critical in health behaviour change [46]. Overall, our study themes are in line with the self-determination theory which overlays emphasis on autonomy–independent decision making, competence–knowledge, skills and self-efficacy, and relatedness–social connections and networks as key determinants of intrinsic motivation that are a desirable bedrock for health behaviour change [32–34]. Indeed, across all behaviours and themes, personal competence and motivation differentiated participants’ motivation to change, uptake of behaviour, and maintenance of the practice. Our study themes also fit the health belief model constructs with factors such as knowledge, awareness and risk perception contributing to the perceived susceptibility, perceived severity and self-efficacy [48]. The benefits and barriers at the individual and social level influence the perceived benefits and barriers to change. These factors then interact to influence the cues to action.

Our study furthers qualitative evidence on the role of community interventions in healthy lifestyles uptake and highlights the mechanism through which this can be achieved. By building personal competence through education programmes and employing motivational interviewing techniques together with social connectedness, community members may be on the path to abandoning unhealthy behaviours. However, as noted, the interplay of these factors influenced the level of behaviour uptake. For dietary and physical activity behaviours, motivation was majorly intrinsic, participants more knowledgeable and skilled and their networks key in adopting the behaviours. On the other hand, smoking and alcohol behaviours, even with relative knowledge reported, motivation for change was more extrinsic and the social network acted in either direction. Smoking and alcohol behaviours being addictive habits are much harder to change and CHWs need further support to build the competence of community members, influence social networks and spur intrinsic motivation towards a gradual process of change. For stress, although motivation was intrinsic, there were knowledge gaps regarding stress reduction techniques as this had not been a key focus of the community intervention [28] and CHWs will need further empowerment on this as an emerging CVD risk factor in the community. The above factors notwithstanding, accessibility and resources remain key and programmes should innovatively work with community members to address these barriers. Indeed, the role of opportunity in behaviour change as advanced by the Capability, Opportunity and Motivation Behaviour (COM-B) model [49] remains key to empowering communities to adopt desired changes. Beyond these factors and as emphasised by the self-determination theory [32, 34], the quality of the intervention and its quantity including reinforcement mechanisms are important. Insights from understanding the uptake of lifestyle practices should guide planning and design of community programmes with an emphasis on removing barriers and enhancing facilitators especially building on the intermediate motivating factors while considering individual needs and expectations. Within the SPICES project, there is further re-enforcement of messages including through more channels such as using information, education and communication materials. The project had previously encouraged more group activities which became unfeasible during the COVID-19 restrictions. Also, moving forward, training for CHWs now incorporate stress reduction techniques and continued tailoring of intervention to community groups.

We conducted this study two months after the COVID-19 total lockdown restrictions in Uganda had started to be eased and it followed a five-months-no-intervention period which impacted uptake of practices and overall programme [50]. Since the intervention was wholly delivered, it is hard to tell which components of it were most supportive. There was also possibility of social desirability bias, however, the research team was not known to most community members and contact was made directly without involving CHWs. The interview guide was also designed with more general questions at the beginning and reference to the ongoing project work was only made at the end. Otherwise, the study delved into an in-depth exploration of uptake motivations of healthy lifestyle behaviours beyond diet and physical activity which most previous studies have focused on. This study provides important evidence to guide tailoring of community interventions aimed at uptake of healthy CVD prevention behaviours in resource-limited settings. A future assessment will focus on adherence and maintenance of the adopted behaviours which was beyond the scope of this study and quantitatively evaluate the levels of uptake of the different behaviours.

## Conclusions

This study found variations in uptake of healthy lifestyle practices for CVD prevention with most changes reported for dietary behaviour and physical activity influenced by more intrinsic motivation, higher competence and positive social influence. On the other hand, participants were slow in making changes in alcohol consumption, smoking behaviours and stress reduction due to differences in motivation, competence, and social influence. The barriers to uptake of healthy lifestyle practices were related to accessibility challenges, limited resources, limited capability or skills, low risk perception and effects of COVID-19. In contrast, the facilitators of practices’ uptake were knowledge, personal determination to change, integration of behaviour into lifestyle and social support. Insights from understanding the uptake of lifestyle practices should guide planning and design of community programmes with an emphasis on removing barriers and enhancing facilitators while considering individual needs and expectations.

## Supporting information

S1 File Interview guide: In-depth interview guide. (DOCX)
