# Supplementary material for: Understanding factors influencing uptake of healthy lifestyle practices among adults following a community cardiovascular disease prevention programme in Mukono and Buikwe districts in Uganda: A qualitative study
Source: PLoS One. 2022 Feb 17;17(2):e0263867. doi: 10.1371/journal.pone.0263867 (PMC8853581; doi:10.1371/journal.pone.0263867)
Supplement: S1 File — (DOCX) [file pone.0263867.s001.docx]

**Understanding factors influencing uptake of healthy lifestyle practices among adults following a community cardiovascular disease prevention programme in Mukono and Buikwe districts in Uganda: a qualitative study**

**In-depth interview guide**

1. I would like to ask you about cardiovascular diseases. Have you heard about these? If so, give me some examples? What is your source of information? (probe: healthcare worker, CHW, TV, others). How can we prevent CVDs? (probe: diet, exercise, smoking, alcohol, salt reduction, maintaining a healthy weight, stress control, regular checkup).

2. Among these practices, what are you currently doing to prevent CVDs? (probe: diet, exercise, smoking, alcohol, maintaining a healthy weight, salt reduction, stress control, regular checkup). When did you start doing this practice? (probe for the practices and when they started). Why did you choose to undertake these measures? (probe for: knowledge, confidence, motivation, other people). How did you find it to undertake these measures? (probe for: was it easy or difficult to change?).

3. How are you able to keep up with these measures to prevent CVDs? What keeps you going? What support is available to you?

4. Have you experienced any benefits ever since you started implementing these measures? Tell me about these benefits? How about any negative effects?

5. Are you confident you can sustain the measures for a long time? Why? If not confident, what can increase your confidence?

6. Are there moments when you have been unable to keep up with the above measures? What moments were those and why did this happen?

7. What aren’t you able to do now that would reduce your risk for cardiovascular diseases? (probes: diet, physical activity, smoking, alcohol use, salt reduction, maintaining a healthy weight, stress control, regular checkup (also consider inter heart behaviours while probing and note whether change occurred or not)). Why aren’t you in position to do this? How ready are you currently to make the change?

8. What can be done to increase your likelihood of undertaking this change (s) in behavior?

9. Have you ever been visited by a community health worker to discuss CVD prevention? How was his/her visit and what did he/she do? What is your perception of their skills and work? What do you think they can do differently?

10. Have you received CVD prevention information through other ways in your community? Tell me about these?

Collect the following socio-demographic characteristics

1. Sex.
2. Age.
3. Education level.
4. Occupation.
5. Marital status.

***Thank you very much for your time.***
